# Face perception across the adult lifespan: evidence for age-related changes independent of general intelligence

**DOI:** 10.1080/02699931.2021.1901657

**Published:** 2021-03-18

**Authors:** Hannah L. Connolly, Andrew W. Young, Gary J. Lewis

**Affiliations:** a Department of Psychology, Royal Holloway, University of London, Egham, England; b Department of Psychology, University of York, Heslington, England

**Keywords:** Face emotion recognition, face identity recognition, general intelligence, age, individual differences

## Abstract

It is well-documented that face perception – including facial expression and identity recognition ability – declines with age. To date, however, it is not yet well understood whether this age-related decline reflects face-specific effects, or instead can be accounted for by well-known declines in general intelligence. We examined this issue using a relatively large, healthy, age-diverse (18-88 years) sample (*N* = 595) who were assessed on well-established measures of face perception and general intelligence. Replicating previous work, we observed that facial expression recognition, facial identity recognition, and general intelligence all showed declines with age. Of importance, the age-related decline of expression and identity recognition was present even when the effects of general intelligence were statistically controlled. Moreover, facial expression and identity ability each showed significant unique associations with age. These results indicate that face perception ability becomes poorer as we age, and that this decline is to some extent relatively focal in nature. Results are in line with a hierarchical structure of face perception ability, and suggest that age appears to have independent effects on the general and specific face processing levels within this structure.

## Introduction

Face perception encompasses a range of abilities that are necessary for successful everyday interactions (Bruce & Young, 2012). Among these abilities, the perception of expression and identity are of critical importance. Accurate perception of facial expressions is essential for appropriate responses to the subtle and rapid changes in a person’s demeanour and emotional state, whilst accurate identification of others via their face enables social interactions to be appropriately adjusted based on prior knowledge of and previous encounters with that individual (Young, 2018).

Of note, then, a substantial body of research has consistently reported a decline in face perception abilities with age. In the context of facial expression recognition, a meta-analysis of 28 datasets (total *N* = 1667) reported age-related decline in face emotion recognition that was evident across categories of emotions (Ruffman, Henry, Livingston, & Philips, 2008). In a sample of 607 participants (18-84 years) who were tested on facial and vocal expression recognition, older participants were shown to be less accurate across emotions (Mill, Allik, Realo, & Valk, 2009). In a sample of 482 participants (20-89 years), participants in their 30’s, 40’s and 50’s showed equivalent accuracy in expression recognition, but a linear decline was seen to emerge from 60 years of age onwards, and further declines were particularly noticeable for participants in their 70’s and 80’s (West et al., 2012). In a large study (*N* = 7230, 18–75 years), Sasson and colleagues observed a deficit for older adults’ expression recognition across all tested emotions (Sasson et al, 2010). Another sample (*N* = 9546, 10–85 years) observed age-related deficits in emotion sensitivity (i.e. discriminating between the intensity of two expressions) (Rutter et al., 2019). Finally, a very large community sample (*N* = 100,257) reported age-related deficits on an emotion recognition task involving composite expressions in a sample of individuals who ranged from younger than 15 years of age to older than 60 years, with the older groups performing worse than their younger counterparts (Olderbak, Wilhelm, Hildebrandt, & Quoidbach, 2019).

In the context of facial identity recognition, age-related changes have also been noted. In a sample of 448 participants (18-88 years), Hildebrandt and colleagues (2010) observed considerable age-related performance decrements across three aspects of identity recognition: face memory (e.g. immediate and delayed recognition of learned faces), face perception (e.g. part-whole matching tasks), and speed of face identity matching (e.g. matching of faces from different viewpoints). Decrements were strongest for the speed of face identity matching (showing a linear decrease beginning in the early 30’s) but were also apparent for memory (the late 40’s) and perception (the 60’s). Age-related decrements have also been reported for another unfamiliar face matching task (Benton, Eslinger, & Damasio, 1981), and in holistic perception (Boutet & Faubert, 2006). In eyewitness identification paradigms, older adults show lower accuracy on line-up tasks, and a higher rate of false recognition of new faces (Searcy, Bartlett, & Memon, 1999).

This body of work provides strong evidence for an age-related decline in face-related abilities. However, it is not yet known if this decline reflects changes in face perception per se, or instead is simply a reflection of well-known age-related declines in general intelligence (Deary, 2001; Salthouse, 2010). A substantial body of empirical research demonstrates the significant age-related declines observed in the domains of reasoning, spatial visualisation, verbal memory and perceptual speed, with vocabulary in contrast showing an increase or preservation until approximately age 60 (Salthouse, 2013). The possibility that this general cognitive decline underpins age-related decline in face perception abilities is bolstered by evidence from recent research demonstrating robust links from general intelligence to both expression recognition (Hildebrandt, Sommer, Schacht, & Wilhelm, 2015; Lewis, Lefevre, & Young, 2016; Schlegel & Scherer, 2016) and identity recognition (Wilhelm et al., 2010; Shakeshaft & Plomin, 2015; Connolly, Young, & Lewis, 2019).

A handful of studies have already attempted to address this issue, although typically without a direct measure of general intelligence. In the context of expression recognition, Mill and colleagues (2009) observed that age remained a significant predictor when controlling for education, a proxy for general intelligence (Deary, Strand, Smith, & Fernandes, 2007). West and colleagues (2012) reported the age/expression recognition association even when controlling for processing speed, which is moderately associated with general intelligence (Neisser et al., 1996; Sheppard & Vernon, 2008). Horning, Cornwell, and Davis (2012) used the Raven’s matrices reasoning test as a proxy for fluid intelligence (Engle, Tuholski, Laughlin, & Conway, 1999), and found that whilst this was a significant predictor of recognition of some of the basic emotions, age also remained a significant predictor. Finally, in terms of identity recognition, Hildebrandt and colleagues (2011) reported that age-related differences in memory for faces were still evident after controlling for age-related differences in general cognition, as measured by Raven’s advanced progressive matrices, and two working memory tasks: a rotation span task and a memory updating task.

However, as alluded to above, a crucial caveat to these studies is the measure of intelligence. In most of the larger studies only a proxy for general intelligence has been used, such as years or level of education (Mill et al., 2009; Sasson et al., 2010; Kessels et al., 2014), matrix reasoning (Horning et al., 2012), or processing speed (West et al., 2012). Whilst these variables undoubtedly correlate with general intelligence (e.g. Deary, Der, & Ford, 2001), it is important to note that they fail to fully capture the broad variance of this ability. It is plausible, then, that if a more comprehensive measure was included, it might completely attenuate the association between age and face perception.

### The current study

The current study sought to offer clarity regarding this important issue by leveraging data from a relatively large, age-diverse sample who had been assessed on well-acknowledged measures of face perception and general intelligence. The Cattell Culture-Fair Intelligence Test comprises four nonverbal subtests, and whilst the constructs of fluid and general intelligence have been debated in the field, factor analytic research has shown very strong correlations (*r* = .77-96) between the Cattell test and other more broadly constructed cognitive batteries, e.g. the General Aptitude Test Battery (Johnson, te Nijenhuis, & Bouchard, 2008), indicating a high level of common measurement across these various cognitive batteries. In comparison to single measures of matrix reasoning or processing speed, then, the Cattell test battery better captures the breadth of general intelligence, and is well suited for our specific research question regarding age-related decline.

The face perception measures included a test of emotion recognition involving morphed images to create differing levels of task difficulty (Young et al., 1997, 2002), thus making it sensitive enough to generate a range of scores and thus suitable for individual differences research in our sample of healthy adults. The measures also included the Benton Test of Facial Recognition (Levin, Hamsher, & Benton, 1975). Whilst the Benton test is based entirely on unfamiliar face recognition (Young & Burton, 2018) and there has been debate about the circumstances in which it is useful (Duchaine & Weidenfeld, 2003; Rossion, 2018) it has the advantages of being a widely-used and purely perceptual measure that generates a range of individual differences in performance. Importantly, in light of the fact that we had access to measures of facial expression and identity recognition in the same sample, the current study was able to examine whether these age-related declines showed *unique* associations with age; that is, whether face perception abilities showed a general decline with age, or whether this decline was specific to expression or identity recognition ability.

## Methods

### Participants

The data analysed in this study were collected by the Cambridge Centre for Ageing and Neuroscience (Cam-CAN) (Shafto et al., 2014). The Cam-CAN sample is cross-sectional and age-diverse (aged 18–88 years). Participants completed demographic questionnaires and general cognitive and memory assessments in a home interview. Following an initial assessment, 700 eligible individuals (50 men and 50 women for every age decile) who were MRI-suitable were invited to complete a range of neuroimaging sessions and cognitive–behavioural tasks, including the cognitive measures examined in the current study. Exclusion criteria for non-eligible participants included: low cognitive health (Mini Mental State Exam score of 24 or lower); poor hearing (failing to hear 35 dB at 1000 Hz in either ear); poor vision (below 20/50 on Snellen test); low English language ability (non-native or non-bilingual English speakers); self-reported substance abuse; and serious health conditions that would affect participation (for example, major neurological or psychiatric conditions, current chemo/radiotherapy, or a history of stroke). A total of 656 (291 men) participants were thus recruited and these data form the basis for the analyses reported here.

Participants were next excluded if they showed chance levels of performance on two or more of the cognitive–behavioural tasks, or had not completed all of the cognitive–behavioural tests (see Measures). Participants were also excluded if they were missing age information. This necessitated the exclusion of 61 participants, resulting in a final sample size of 595 (291 men). The mean age of participants was 54.0 years (SD = 18.2, range = 18-88), and ethnicity was as follows: White (*N* = 573), Asian (*N* = 7), Black (*N* = 1), Mixed Race (*N* = 8) and undisclosed (*N* = 6).

### Measures

***Facial expression recognition ability*** was assessed using the Emotion Hexagon test (Young et al., 1997, 2002). This test was created by using a model from the Ekman and Friesen (1976) “Pictures of facial affect” series displaying each of the six basic emotions (anger, disgust, fear, happiness, sadness, and surprise). These prototypical emotion images were then morphed with another basic emotion to form emotional expressions with graded levels of difficulty (expression pairs morphed together consist of happiness-surprise, surprise-fear, fear-sadness, sadness-disgust, disgust-anger, and anger-happiness). Participants were shown faces with either 70% or 90% of the target emotion, and had to make a six-alternative forced-choice response to indicate whether the expression was most like anger, disgust, fear, happiness, sadness, or surprise. There were 20 trials for each of the six emotions, and stimuli were shown for 3 s each. A percentage accuracy score for each of the six emotions was generated for use in subsequent analyses. The six Emotion Expression Recognition sub-scores were significantly associated: *r* ranged from .12 to .46, and all *p* < .003.

***Facial recognition ability*** was assessed using the short-form of the Benton Test of Facial Recognition (Levin, Hamsher, & Benton, 1975), which measures the ability to match pictures of unfamiliar faces. The test consists of 27 trials in which the participant is shown one target face and an array of six faces. The participant has to identify one or more examples of the target face in the array. There may be changes in head orientation or lighting between the target and array faces. Each correct response receives a score of 1, and a total percentage accuracy score was generated for use in subsequent analyses.

***General intelligence*** was assessed using the Cattell Culture Fair Intelligence test (Scale 2 Form A: Cattell, 1973), which contains four nonverbal subtests: Series Completion, Classification, Matrices, and Conditions. Participants are given 3, 4, 3, and 2.5 min, respectively to complete each subtest. The test uses a pen-and-paper approach: the participant is asked to choose a response for each item from multiple response options and to record their response on a corresponding answer sheet. Correct responses are given a score of 1 and the percentage correct for each sub-test was calculated for use in subsequent analyses. The four Cattell Culture Fair Intelligence subtests were significantly associated: *r* ranged from .52 to .63 (all *p* < .001).

### Procedure

Eligible participants attended testing sessions at the Medical Research Council Cognition and Brain Sciences Unit in Cambridge UK. Approximate duration for each of the tasks was as follows: Facial expression recognition: 20 min; Unfamiliar facial identity recognition: 10 min; and General intelligence: 20 min. The facial expression recognition test was presented on a laptop, and the unfamiliar facial recognition and intelligence tests were administered using pen and paper. The majority of participants were comfortable using the laptop for the facial expression task, but if a participant struggled, the researcher pressed the buttons for them in response to their spoken answer. This ensured that the accuracy of a participant’s answer would not be confounded by their computer competency.

## Analysis

### Measurement invariance

As an initial validity check, we tested for measurement invariance separately for the two variables with sufficient number of manifest variables to stably identify a latent factor (General Intelligence: four Cattell subtests; Face Expression recognition: six emotion categories).

#### General intelligence

Firstly, we tested for configural invariance by examining whether the same pattern of freed and fixed parameters held across three defined age groups (Younger Adults: 18–39 (*N* = 153); Middle-aged Adults: 40–64 (*N* = 243); Older Adults: 65+ (*N* = 199)). Model results demonstrated that configural invariance was evident across the three age groups, (*Χ*^2^ (6) = 5.36, *p* = .499; CFI = 1.00; RMSEA = .00).

#### Complete metric (weak factorial) invariance testing: general intelligence

Secondly, metric invariance (i.e. weak factorial invariance) was tested by examining if the factor loadings were equivalent across groups. Model results in aggregate demonstrated evidence for complete metric invariance (*Χ*^2^ (12) = 21.64, *p* = .042; CFI = .98; RMSEA = .06). However, the chi square difference between this model and the configural model was significant, *Χ*^2^ difference (6) = 16.28, *p* = .012, suggesting that the metric model had a significantly worse fit.

#### Partial metric (weak factorial) invariance testing: general intelligence

We thus explored whether metric model fit could be improved by adjusting some model parameters. For this, we inspected the modification indices and in turn allowed the loading of the second Cattell subtest (Classification) to vary across age groups, with the other three subtest loadings remaining constrained to equality across the age groups. We re-ran the metric invariance test with this modification and found this model had excellent fit *(Χ*^2^ (10) = 10.84, *p* = .370; CFI = .99; RMSEA = .02). Moreover, the chi square difference between this partial metric model and the configural model was non-significant, *Χ*^2^ difference (4) = 5.48, *p* = .241.

The results of this invariance testing suggest that the factor structure of general intelligence is equivalent across age groups. However, complete metric invariance was not able to be established, suggesting that at least some of the age group differences in general intelligence reflect variance beyond the general factor level of analysis.

#### Face expression recognition

Secondly, we assessed whether face expression recognition ability was invariant across age. Firstly, configural invariance was established across the three age groups, (*Χ*^2^ (21) = 29.44, *p* = .104; CFI = .98; RMSEA = .05).

#### Complete metric (weak factorial) invariance testing: face expression recognition

Model results in aggregate demonstrated evidence for complete metric invariance (*Χ*^2^ (31) = 51.95, *p* = .011; CFI = .945; RMSEA = .058). However, the chi square difference between this model and the configural model was significant, *Χ*^2^ difference (10) = 22.51, *p* = .013, suggesting that the metric model had a significantly worse fit.

#### Partial metric (weak factorial) invariance testing: face expression recognition

As before, we explored whether the metric model fit could be improved by adjusting some model parameters. For this, we inspected the modification indices and allowed the loading of the Happiness manifest variable to vary, with the other five emotion category loadings remaining equivalent across the age groups. We re-ran the metric invariance test with this partial constraint, and found this model did not have an acceptable fit according to the chi square statistic, *Χ*^2^ (29) = 44.78, *p* = .031, but had acceptable alternative fit indices (CFI = .958; RMSEA = .052). Moreover, the chi square difference between this partial metric model and the configural model was non-significant, *Χ*^2^ difference (8) = 15.33, *p* = .053, although interpreting *p* values close to the nominal threshold should be done with caution.

The results of this invariance testing suggest some relatively modest evidence of metric variance of expression recognition across age groups. Complete weak invariance was not able to be established, suggesting that at least some of the age group difference in expression recognition factor loadings is attributable to measurement bias. However, scale invariance was established for both general intelligence and face expression recognition, so we elected to continue using these measures to assess age differences as per our analysis plan.

## Results

**Figure 1.**
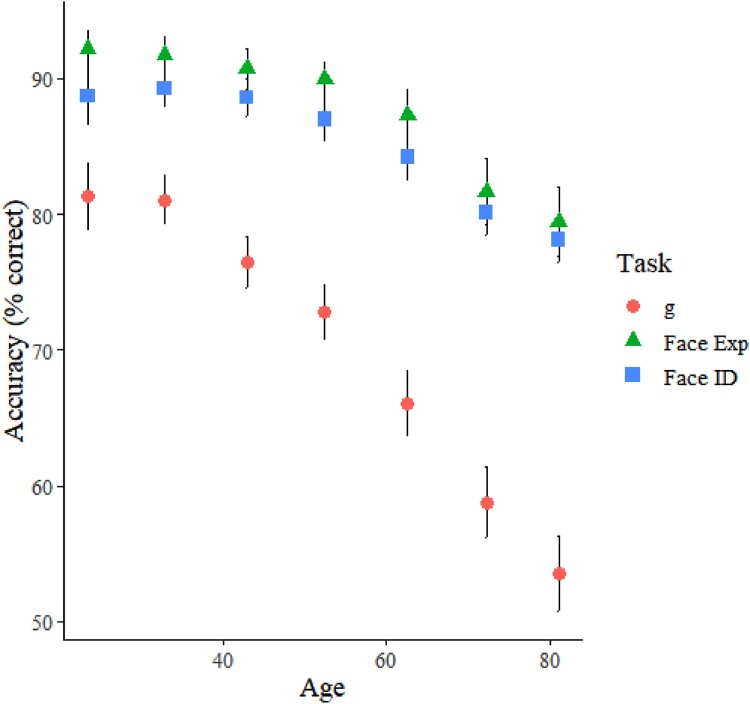
Relationships with age (by decile) for general intelligence (g), facial expression recognition ability (Face Exp), and facial identity recognition ability (Face ID).

Descriptive statistics are detailed in Table 1. Inter-correlations between study variables are detailed in Table 2. Facial expression recognition showed strong positive correlations with general intelligence and facial identity recognition. Age was negatively associated with expression and identity recognition, and with general intelligence. These age relationships are also illustrated in Figure 1.

**Table 1.** Descriptive statistics of facial expression recognition ability (Face Exp), facial identity recognition ability (Face ID), age, and general intelligence.

	Mean	SD	Min	Max	Skew	Kurtosis
Age	54.01	18.16	18	88	−.04	−1.12
Face Exp	87.43	9.83	26.09	95.65	−1.36	1.75
Face ID	85.07	8.50	49.17	100.00	−.36	−.50
General Intelligence	69.58	14.48	62.96	100.00	−.56	−.14

**Table 2.** Zero-order correlations between measures of facial expression recognition ability (Face Exp), facial identity recognition ability (Face ID), age, and general intelligence.

	Face ID	General Intelligence	Age
Face Exp	.39 (.22)	.52	−.44 (−.15)
Face ID		.42	-−46 (−.27)
General Intelligence			−.66

Note: Values in parentheses reflect correlations when controlling for general intelligence. All *p* < .001.

### Regression analysis

The regression analyses then enabled us to test our research question of whether the age-related decline in expression recognition or identity recognition was independent from the decline observed in general intelligence.

In the first regression model, expression recognition was entered as the dependent variable, and general intelligence, age, sex, and identity recognition were all entered as predictors in the same step. The coefficients indicated that each of these variables was a significant and unique predictor of expression recognition. The full results of this regression analysis are shown in Table 3. Note that the coefficient for sex reflects face expression recognition scores being significantly higher for women. This finding is further analysed and discussed below.

**Table 3.** Multiple regressions predicting facial expression recognition ability from age, sex, facial identity recognition ability (Face ID), and general intelligence.

Independent Variables	*β*	Sig
Age	**−**.10	.04
Sex	.14	<.001
Face ID	.18	<.001
General Intelligence	.39	<.001

In the second regression model, identity recognition was the dependent variable, and general intelligence, age, sex, and expression recognition were all entered as predictors. In this case, the coefficients for general intelligence, age, and expression recognition all suggested unique influence of these variables on identity recognition, but sex was not a significant predictor. The full results of this analysis are shown in Table 4.

**Table 4.** Multiple regressions predicting facial identity recognition ability from age, sex, facial expression recognition ability (Face Exp), and general intelligence.

Independent Variables	*β*	Sig
Age	**−**.29	<.001
Sex	.02	.63
Face Exp	.20	<.001
General Intelligence	.13	.01

**Figure 2.**
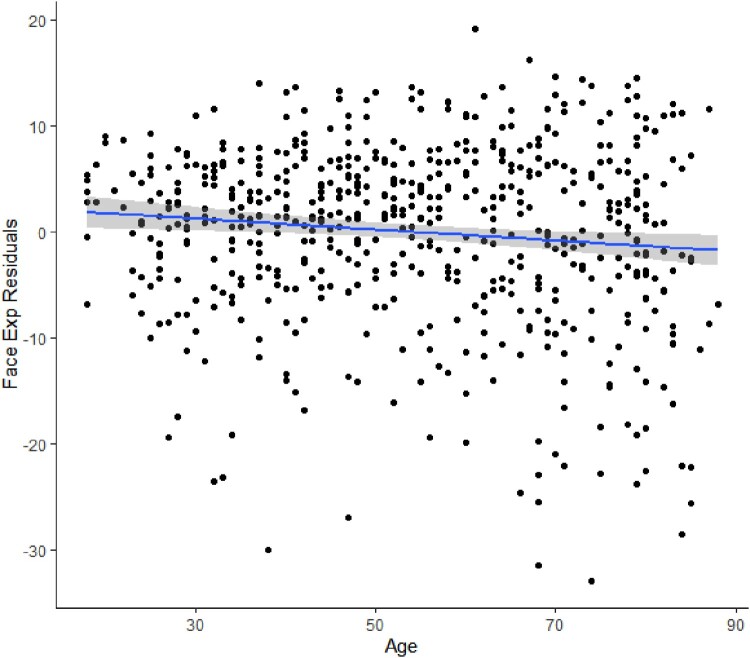
Relationships of age with face expression (Face Exp) recognition residuals, showing the age decline of Face Exp when controlling for general intelligence.

The age declines in face perception abilities when controlling for general intelligence are further illustrated through plotting of the residuals, and are shown in Figures 2 (expression recognition) and 3 (identity recognition).

**Figure 3.**
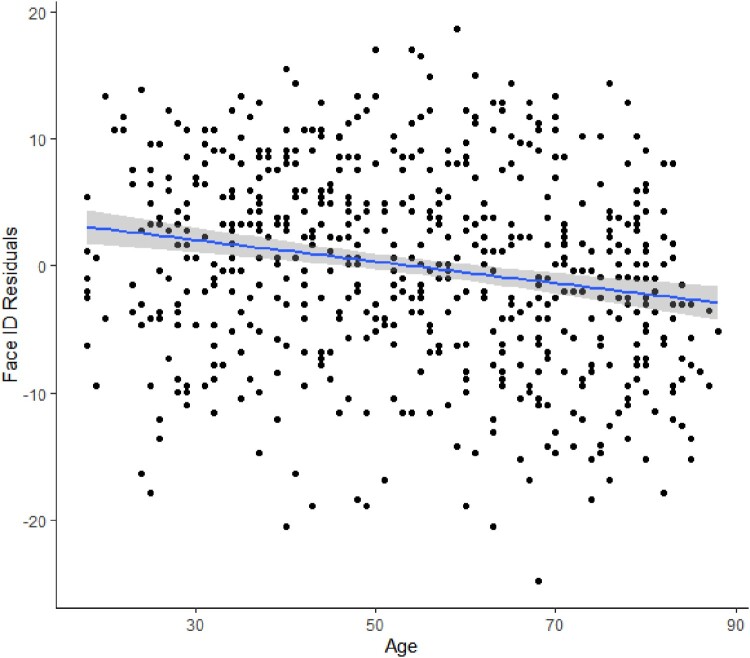
Relationships of age with face identity (Face ID) recognition residuals, showing the age decline of Face ID when controlling for general intelligence.

### Mediation analyses

**Figure 4.**
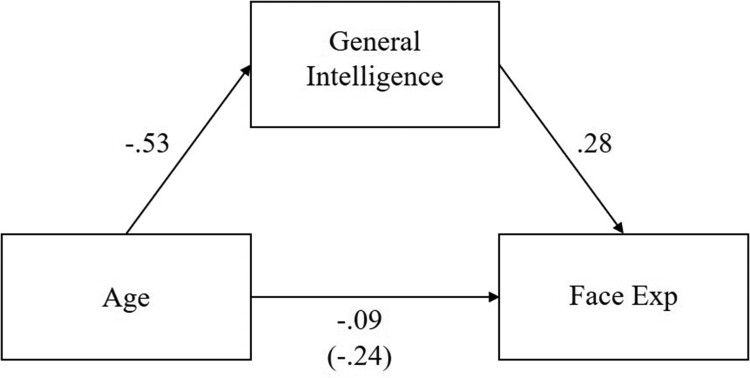
Mediation model of age, general intelligence, and facial expression recognition ability. Note: All standardised coefficients are significant at *p* < .001. The value in parentheses is the relationship between age and facial expression recognition before general intelligence was taken into account.

**Figure 5.**
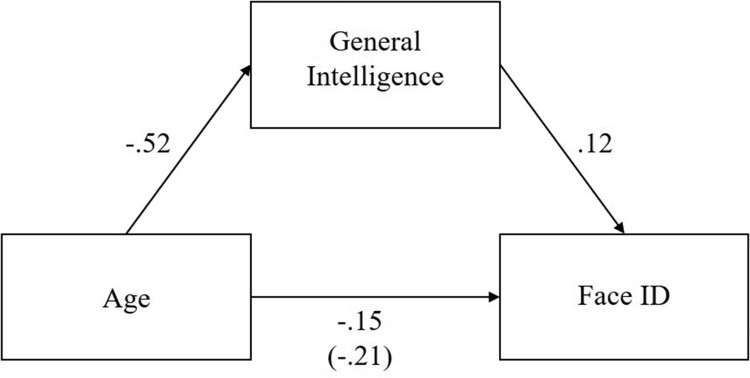
Mediation model of age, general intelligence, and facial identity recognition ability. Note: All standardised coefficients are significant at *p* < .001. The value in parentheses is the relationship between age and facial identity recognition before general intelligence was taken into account.

We note from the linear regressions presented above that both age and general intelligence are significant unique predictors of facial expression recognition and facial identity recognition ability. We formally tested for mediation effects using a path analysis approach implemented in the R package “lavaan” (Rosseel, 2012). We tested one plausible model, whereby age was the independent variable, general intelligence the mediating variable, and facial expression or facial identity recognition as the respective dependent variable. While this arguably reflects the most theoretically plausible causal model, other possible pathway models exist, and as such we advise caution when interpreting these paths. The mediated relationships are presented in Figures 4 and 5.

Across the two models, age significantly predicted facial expression (*β* = −.09, CI = [−.14, -.04], *p* < .001), and facial identity recognition (*β* = −.15, CI = [−.19, -.11], *p* < .001), even when general intelligence was included in the model. The indirect effect of age through general intelligence was a significant predictor of facial expression (*β* = −.15, CI = [−.18, −.11], *p* < .001), and facial identity recognition (*β* = −.06, CI = [-.09, -.03], *p* < .001), indicating that general intelligence was a partial mediator of the effect between age and facial expression, and between age and facial identity recognition.

### Subsidiary analyses

**Figure 6.**
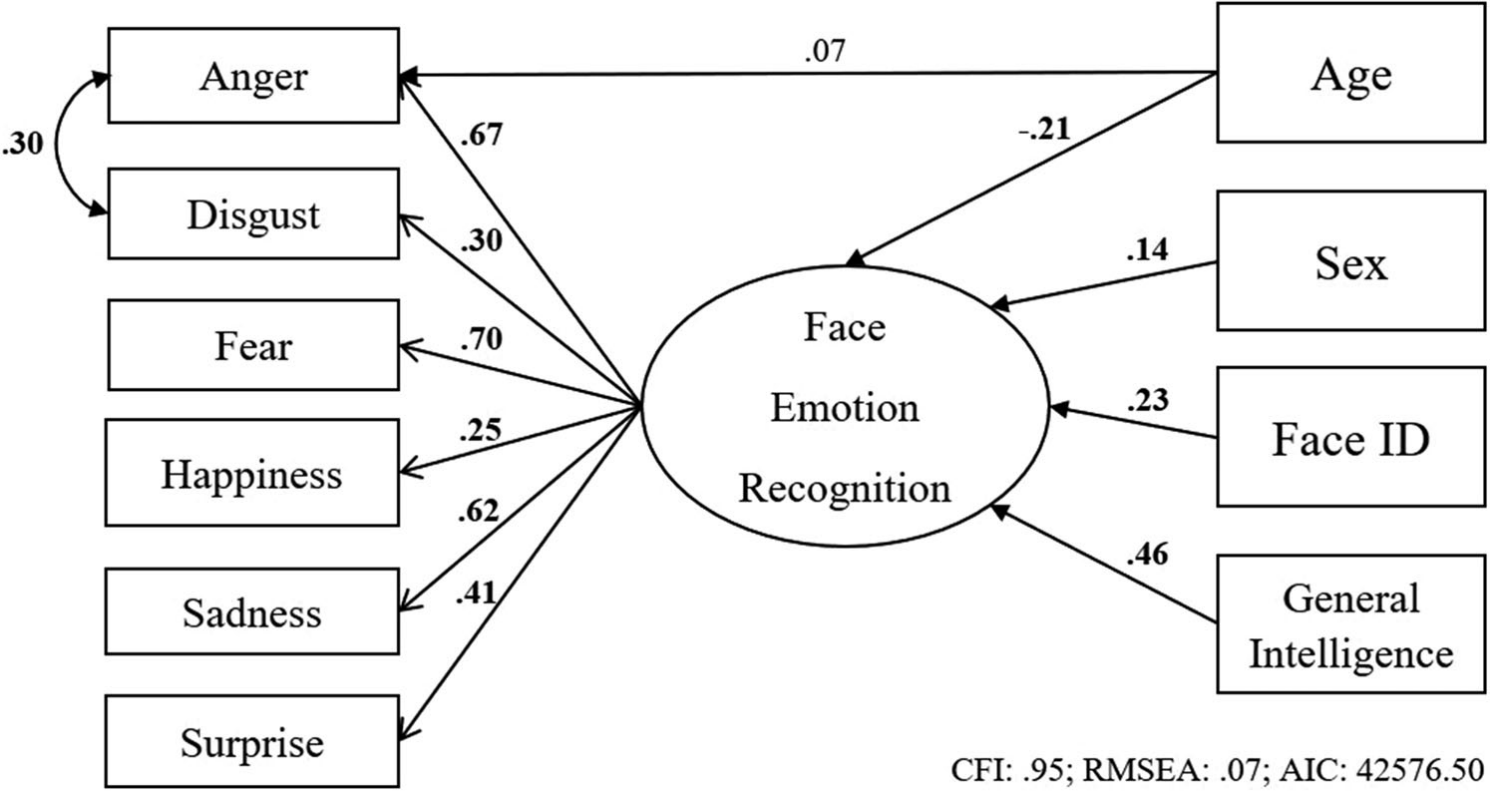
Graphical representation of a model predicting facial emotion expression recognition ability from age, sex, facial identity recognition ability (Face ID), and general intelligence, with age also directly predicting anger recognition.

Firstly, we ran an exploratory analysis to examine whether age-related decline in facial expression recognition ability instead reflected worse performance on one or more particular emotion categories. If we had directly correlated age with each of the specific emotion recognition variables, any observed association could reflect specific emotion recognition variance, general factor variance, or both. To avoid this situation we used a structural equation modelling approach, which allowed us to simultaneously estimate the relationship between age and emotion recognition, both at the general factor level and the specific emotion level (i.e. the residual variance of the specific emotion recognition variables). We fitted six structural equation models (age was a predictor of only one specific emotion recognition variable in these models; to have estimated all specific emotion recognition paths would have led to an under-identified model) in which we estimated the effect of age on the general emotion factor, and the residual variance of each emotion directly predicted by age, over and above the general factor. We also included the variables of general intelligence, sex, and face identity recognition. In line with previous modelling of this dataset (Connolly, Young, & Lewis, 2019), we allowed anger and disgust to covary, given their close relationship. An example of this model (with age predicting anger) is shown in Figure 6 above. Bolded values indicate significance at *p* < .05, and fit indices are presented below the model.

For each of the six models, the loadings of age onto the general emotion factor and the individual emotion are shown in Table 5 below. In all models (excluding the one with the pathway from age to fear), age significantly negatively predicted the general emotion factor (−.17 to −.21, *p* ≤ .002), as would be expected from results presented above. Full model results are presented in the Appendix.

**Table 5.** Path loadings and significance levels of the six structural equation models, and their corresponding fit indices.

Emotion	Age -> Emotion Variable	Sig	Age -> Latent ERA	Sig	CFI	RMSEA
Anger	.07	.192	**−.21**	<.001	.95	.067
Disgust	.**25**	<.001	**−**.**20**	<.001	.96	.057
Fear	**−**.**17**	<.001	**−**.09	.112	.96	.062
Happiness	.**13**	.016	**−**.**20**	<.001	.95	.065
Sadness	.04	.451	**−**.**20**	<.001	.95	.068
Surprise	**−**.**11**	.032	**−**.**17**	.002	.95	.066

Note: ERA = Emotion Recognition Ability.

The unique associations between age and each of the specific emotion recognition abilities were more mixed. Fear and surprise were negatively predicted by age, disgust and happiness were positively predicted by age, and anger and sadness showed no significant association with age over and above the variance accounted for by the general factor.

Secondly, whilst not of primary importance to the present study, the observation of a significant sex difference in favour of women for emotion recognition ability was deemed sufficiently important for us to present here for issues of replication, and thus was further explored in a subsidiary analysis. We tested whether this effect was evident across all of the emotions, or if it reflected specific emotional categories, in light of recent work noting a selective female advantage for recognising facial disgust (Connolly, Lefevre, Young, & Lewis, 2020). We subjected the data to six *t* tests, correcting for multiple comparisons (Bonferroni-corrected: adjusted *α* = .0083). There was a significant difference in favour of women on recognition of disgust (*t*(593)=−3.22, *p* = .001, Cohen’s *d* = .26, [CI95%:.10-.43]) (female M = 88.59, SD = 15.98; male M = 83.83, SD = 19.94) and in recognition of happiness (*t*(593) = −3.39, *p* = .001, Cohen’s *d* = .28, [CI95%: .12-.44]) (female M = 98.17, SD = 4.17; male M = 96.68, SD = 6.37).

## Discussion

A number of studies have reported age-related declines in facial expression and identity recognition abilities. However, to date, it has not been well understood if these declines reflect independent expression and identity effects, a more general face-specific effect, or simply the manifestation of the well-acknowledged decline in general intelligence observed across the lifespan (Deary, 2001). Moreover, as we have noted in the Introduction, studies to date have not used sufficiently broad measures of intelligence to fully answer this question. To address both the theoretical question and this methodological issue here, we used a relatively large cross-sectional sample of individuals ranging from 18 to 88 years of age who were assessed on widely used tests of face perception ability, and most importantly for current purposes, a comprehensive measure of general intelligence.

Expression recognition ability, identity recognition ability, and general intelligence were all negatively related to age, such that older individuals scored more poorly. Of importance, age was a significant predictor of both expression and identity recognition ability, even when general intelligence was statistically controlled, indicating that these age-related declines are not fully accounted for by the known decline in general intelligence. Indeed, the mediation analyses indicated that general intelligence was a partial mediator of the effect of age on facial expression/identity recognition. These findings are consistent with previous work that found age remained a significant predictor after controlling for proxies of general intelligence (Horning et al., 2012; West et al, 2012). However, given that the current study used a comprehensive measure of general intelligence as opposed to a proxy measure, our results here support this finding in stronger and more concrete terms.

Whilst it is clear that different measures of face perception will often correlate with each other, such correlations are typically able to account for a maximum of around 25% of the variance across face tasks (Connolly et al., 2020; McCaffery et al., 2018; Verhallen et al., 2017). Consistent with this, the intercorrelation between the face perception variables tested here (0.39) accounted for some 15% of variance and each measure showed significant unique associations with age. This suggests that whilst the face variables themselves covary in a way that is consistent with the idea of a general factor underlying different aspects of face perception (Verhallen et al., 2017), their respective declines are to some extent independent of one another, and not solely attributable to a general overall decline in face processing ability.

This finding of independent associations of expression recognition and identity recognition to age is consistent with previous related work suggesting multiple levels of individual differences underlying face perception ability, including general intelligence, general face-specific processing, and expression- and identity-specific processes (Lewis et al., 2016; Connolly et al., 2020). This suggests then, that the effect of age may operate differently at these various levels of processing, and that at least some of the age-related decline acts upon the expression- and identity-specific level, resulting in the independent age associations that we have observed in the current study.

Our findings are also consistent with the age-related decline often observed in broader domains such as social cognition. For example, Maylor and colleagues (2002) reported a significant negative association between theory of mind (ToM) and age, when controlling for vocabulary, processing speed, and executive functioning. More recently, Baksh and colleagues (2018) developed a test of social cognition that assessed both cognitive and affective ToM and inter- and intrapersonal understanding of social norms, and found that whilst scores on this test declined with increasing age, they were not correlated with either verbal or reasoning ability. These results suggest, then, that in line with our current face perception results, social cognition may also show a somewhat independent age-related decline from general intelligence. Further studies will be able to offer further insight into the extent of this putative independence.

Our subsidiary analyses revealed two interesting observations. Firstly, while age was negatively associated with emotion recognition ability in aggregate terms, we observed more nuanced results when assessing the age relationship with specific emotions (while controlling for general factor variance in emotion recognition ability). Specifically, we saw that fear and surprise were negatively associated with age, whereas disgust and happiness were positively associated with age. These results suggest that while age might impact on general emotion recognition ability, some degree of preservation or even improvement on recognition of specific emotions is apparent, perhaps via a shift in processing strategies. These findings are reflected in some previous work on emotion recognition and ageing. For example, several studies have highlighted the relative preservation of happiness and disgust recognition in older participants (Calder et al., 2003; Ruffman et al., 2008). More generally, these results suggest that relationships between age and specific emotion recognition abilities may be obscured when examining only the general emotion recognition factor, and highlights a need to partial out the general factor variance from unique variance when trying to better understand the complex relationship between age and emotion recognition ability.

Secondly, we observed a significant female advantage for recognising facial disgust and happiness, suggesting that the overall emotion recognition difference was being primarily driven by women’s more accurate recognition of these two discrete emotions.

Some limitations of the current study are worth noting. Firstly, the design is cross-sectional, with participants being tested at only one time point, and therefore cohort effects between different generations may be a source of bias. The environments in which our younger and older participants developed are likely to vary greatly, with large differences in cultural norms and quality and quantity of healthcare, nutrition, and education, amongst other variables. Indeed, in intelligence research, the phenomenon of cognitive test scores increasing across generations has been widely established (Flynn, 1987). However, it has also been noted that within-cohort variation can be almost as large as that between different cohorts (Salthouse, 2014a), suggesting that age-related differences in cognition cannot be wholly accounted for by cohort differences. Additionally, whilst longitudinal designs have often reported positive test effects whereby participants show benefits of having had prior test experience, quasi-longitudinal designs have reported almost identical age trends to cross-sectional studies (Salthouse, 2014b). This suggests, then, that longitudinal designs may underestimate the negative age-related change in cognition, and that cross-sectional results may be closer to estimating the true magnitude of age-related decline. Given this, then, future studies may benefit from employing longitudinal designs to corroborate the findings of cross-sectional studies.

Secondly, we must consider the likely bias induced by self-selection. The individuals comprising the current sample were recruited as part of a larger study in which participants had to attend multiple testing sessions involving MRI (magnetic resonance imaging) and MEG (magnetoencephalography) measures. Being willing and physically able to attend these sessions and complete a variety of cognitive and neural tasks suggests a certain level of motivation. In addition, in order to be eligible to take part in the neuroimaging stage of the study, the individuals had to be healthy with no serious cognitive impairment, psychiatric disorders, difficulties with vision or hearing, or evidence of substance abuse. Given the extensive cognitive and physical screening of our participants before testing (Shafto et al., 2014), the observed age-related declines in face perception are unlikely to be due to comprehension or sensory difficulties. Therefore, selected participants are likely to represent the higher end of the typical continuum in the general population, and this may be especially true for the older participants. It should be noted, though, that this source of bias would likely have led to an underestimate of the age effects observed here.

Finally, we established configural invariance of general intelligence and expression recognition, but only established partial metric invariance for these two variables. This suggests only partially equivalent factor loadings across the age groups, and that at least some of the age effect observed for intelligence and expression recognition may be accounted for by measurement bias. We suggest that future studies of expression recognition and general intelligence should seek to establish complete metric invariance of their chosen measures to ensure accurate interpretation of any age effects observed.

### Conclusions

In summary, the current study observed age-related declines in facial expression and facial identity recognition abilities in a relatively large, healthy, age-diverse sample. Importantly, these declines were not fully explained by controlling for the known age-related decline in general intelligence, even when this was thoroughly measured. Furthermore, the declines in expression and identity recognition were to some extent domain-specific, and not merely a function of broader face processing age-related difficulties.

## Supplementary Material

Supplementary_Material

## Data Availability

Face perception across the adult lifespan: Evidence for age-related changes independent of general intelligence. The data analysed in this study were collected by the Cambridge Centre for Ageing and Neuroscience (Cam-CAN) (Shafto et al., 2014) and are available upon application from the original authors at www.cam-can.org.

## References

[CIT0001] Baksh, R. A., Abrahams, S., Auyeung, B., MacPherson, S. E., & van den Bos, R. (2018). The Edinburgh social cognition test (ESCoT): Examining the effects of age on a new measure of theory of mind and social norm understanding. *PloS One*, *13*(4), e0195818. 10.1371/journal.pone.0195818 29664917 PMC5903589

[CIT0002] Benton, A. L., Eslinger, P. J., & Damasio, A. R. (1981). Normative observations on neuropsychological test performances in old age. *Journal of Clinical and Experimental Neuropsychology*, *3*(1), 33–42. 10.1080/01688638108403111 7276195

[CIT0003] Boutet, I., & Faubert, J. (2006). Recognition of faces and complex objects in younger and older adults. *Memory & Cognition*, *34*(4), 854–864. 10.3758/BF03193432 17063916

[CIT0004] Bruce, V., & Young, A. W. (2012). *Face perception*. Psychology Press.

[CIT0005] Calder, A. J., Keane, J., Manly, T., Sprengelmeyer, R., Scott, S., Nimmo-Smith, I., & Young, A. W. (2003). Facial expression recognition across the adult life span. *Neuropsychologia*, *41*(2), 195–202. 10.1016/S0028-3932(02)00149-5 12459217

[CIT0006] Cattell, R. B. (1973). *Culture-fair intelligence test*. Institute for personality and ability testing.

[CIT0007] Connolly, H. L., Lefevre, C. E., Young, A. W., & Lewis, G. J. (2020). Emotion recognition ability: Evidence for a supramodal factor and its links to social cognition. *Cognition*, *197*, 104166. 10.1016/j.cognition.2019.104166 31951857

[CIT0008] Connolly, H. L., Young, A. W., & Lewis, G. J. (2019). Recognition of facial expression and identity in part reflects a common ability, independent of general intelligence and visual short-term memory. *Cognition and Emotion*, *33*(6), 1119–1128. 10.1080/02699931.2018.1535425 30336725

[CIT0009] Deary, I. J. (2001). *Intelligence: A very short introduction*. Oxford University Press.

[CIT0010] Deary, I. J., Der, G., & Ford, G. (2001). Reaction times and intelligence differences: A population-based cohort study. *Intelligence*, *29*(5), 389–399. 10.1016/S0160-2896(01)00062-9

[CIT0011] Deary, I. J., Strand, S., Smith, P., & Fernandes, C. (2007). Intelligence and educational achievement. *Intelligence*, *35*(1), 13–21. 10.1016/j.intell.2006.02.001

[CIT0012] Duchaine, B. C., & Weidenfeld, A. (2003). An evaluation of two commonly used tests of unfamiliar face recognition. *Neuropsychologia*, *41*(6), 713–720. 10.1016/S0028-3932(02)00222-1 12591028

[CIT0013] Ekman, P., & Friesen, W. V. (1976). *Pictures of facial affect*. Consulting Psychologists Press.

[CIT0014] Engle, R. W., Tuholski, S. W., Laughlin, J. E., & Conway, A. R. (1999). Working memory, short-term memory, and general fluid intelligence: A latent-variable approach. *Journal of Experimental Psychology: General*, *128*(3), 309–331. 10.1037/0096-3445.128.3.309 10513398

[CIT0015] Flynn, J. R. (1987). Massive IQ gains in 14 nations: What IQ tests really measure. *Psychological Bulletin*, *101*(2), 171–191. 10.1037/0033-2909.101.2.171

[CIT0016] Hildebrandt, A., Sommer, W., Herzmann, G., & Wilhelm, O. (2010). Structural invariance and age-related performance differences in face cognition. *Psychology and Aging*, *25*(4), 794–810. 10.1037/a0019774 20822255

[CIT0017] Hildebrandt, A., Sommer, W., Schacht, A., & Wilhelm, O. (2015). Perceiving and remembering emotional facial expressions—A basic facet of emotional intelligence. *Intelligence*, *50*, 52–67. 10.1016/j.intell.2015.02.003

[CIT0018] Hildebrandt, A., Wilhelm, O., Schmiedek, F., Herzmann, G., & Sommer, W. (2011). On the specificity of face cognition compared with general cognitive functioning across adult age. *Psychology and Aging*, *26*(3), 701–715. 10.1037/a0023056 21480718

[CIT0019] Horning, S. M., Cornwell, R. E., & Davis, H. P. (2012). The recognition of facial expressions: An investigation of the influence of age and cognition. *Aging, Neuropsychology, and Cognition*, *19*(6), 657–676. 10.1080/13825585.2011.645011 22372982

[CIT0020] Johnson, W., te Nijenhuis, J., & Bouchard, T. J., Jr. (2008). Still just 1g: Consistent results from five test batteries. *Intelligence*, *36*(1), 81–95. 10.1016/j.intell.2007.06.001

[CIT0021] Kessels, R. P., Montagne, B., Hendriks, A. W., Perrett, D. I., & de Haan, E. H. (2014). Assessment of perception of morphed facial expressions using the emotion recognition task: Normative data from healthy participants aged 8–75. Journal of Neuropsychology, *8*(1), 75–93. 23409767 10.1111/jnp.12009

[CIT0022] Levin, H. S., Hamsher, K. D. S., & Benton, A. L. (1975). A short form of the test of facial recognition for clinical use. *The Journal of Psychology*, *91*(2), 223–228. 10.1080/00223980.1975.9923946

[CIT0023] Lewis, G. J., Lefevre, C. E., & Young, A. W. (2016). Functional architecture of visual emotion recognition ability: A latent variable approach. *Journal of Experimental Psychology: General*, *145*(5), 589–602. 10.1037/xge0000160 26986040

[CIT0024] Maylor, E. A., Moulson, J. M., Muncer, A. M., & Taylor, L. A. (2002). Does performance on theory of mind tasks decline in old age? *British Journal of Psychology*, *93*(4), 465–485. 10.1348/000712602761381358 12519529

[CIT0025] McCaffery, J., Robertson, D. I., Young, A. W., & Burton, A. M. (2018). Individual differences in face identity processing. *Cognitive Research: Principles and Implications*, *3*(21), 1–15. 10.1186/s41235-018-0112-9 30009251 PMC6019420

[CIT0026] Mill, A., Allik, J., Realo, A., & Valk, R. (2009). Age-related differences in emotion recognition ability: A cross-sectional study. *Emotion*, *9*(5), 619–630. 10.1037/a0016562 19803584

[CIT0027] Neisser, U., Boodoo, G., Bouchard, T. J. Jr, Boykin, A. W., Brody, N., Ceci, S. J., Halpern, D. F., Loehlin, J. C., Perloff, R., Sternberg, R. J., & Urbina, S. (1996). Intelligence: Knowns and unknowns. *American Psychologist*, *51*(2), 77–101. 10.1037/0003-066X.51.2.77

[CIT0028] Olderbak, S., Wilhelm, O., Hildebrandt, A., & Quoidbach, J. (2019). Sex differences in facial emotion perception ability across the lifespan. *Cognition and Emotion*, *33*(3), 579–588. 10.1080/02699931.2018.1454403 29564958

[CIT0029] Rosseel, Y. (2012). Lavaan: An R package for structural equation modeling and more. Version 0.5–12 (BETA). *Journal of Statistical Software*, *48*(2), 1–36. 10.18637/jss.v048.i02

[CIT0030] Rossion, B. (2018). Normative accuracy and response time data for the computerized Benton facial Recognition Test (BFRT-c). *Behavior Research Methods*, *50*(6), 2442–2460. 10.3758/s13428-018-1023-x 29549569

[CIT0031] Ruffman, T., Henry, J. D., Livingstone, V., & Phillips, L. H. (2008). A meta-analytic review of emotion recognition and aging: Implications for neuropsychological models of aging. *Neuroscience & Biobehavioral Reviews*, *32*(4), 863–881. 10.1016/j.neubiorev.2008.01.001 18276008

[CIT0032] Rutter, L. A., Dodell-Feder, D., Vahia, I. V., Forester, B. P., Ressler, K. J., Wilmer, J. B., & Germine, L. (2019). Emotion sensitivity across the lifespan: Mapping clinical risk periods to sensitivity to facial emotion intensity. *Journal of Experimental Psychology. General*, *148*(11), 1993–2005. 10.1037/xge0000559 30777778

[CIT0033] Salthouse, T. A. (2010). Selective review of cognitive aging. *Journal of the International Neuropsychological Society*, *16*(5), 754–760. 10.1017/S1355617710000706 20673381 PMC3637655

[CIT0034] Salthouse, T. A. (2013). Within-cohort age-related differences in cognitive functioning. *Psychological Science*, *24*(2), 123–130. 10.1177/0956797612450893 23319401 PMC3638128

[CIT0035] Salthouse, T. A. (2014a). Why are there different age relations in cross-sectional and longitudinal comparisons of cognitive functioning? *Current Directions in Psychological Science*, *23*(4), 252–256. 10.1177/0963721414535212 25382943 PMC4219741

[CIT0036] Salthouse, T. A. (2014b). Aging cognition unconfounded by prior test experience. *Journals of Gerontology Series B: Psychological Sciences and Social Sciences*, *71*(1), 49–58. 10.1093/geronb/gbu063 PMC4840365 25182845

[CIT0037] Sasson, N. J., Pinkham, A. E., Richard, J., Hughett, P., Gur, R. E., & Gur, R. C. (2010). Controlling for response biases clarifies sex and age differences in facial affect recognition. *Journal of Nonverbal Behavior*, *34*(4), 207–221. 10.1007/s10919-010-0092-z

[CIT0038] Schlegel, K., & Scherer, K. R. (2016). Introducing a short version of the geneva emotion Recognition Test (GERT-S): psychometric properties and construct validation. *Behavior Research Methods*, *48*(4), 1383–1392. 10.3758/s13428-015-0646-4 26416137

[CIT0039] Searcy, J. H., Bartlett, J. C., & Memon, A. (1999). Age differences in accuracy and choosing in eyewitness identification and face recognition. *Memory & Cognition*, *27*(3), 538–552. 10.3758/BF03211547 10355242

[CIT0040] Shafto, M. A., Tyler, L. K., Dixon, M., Taylor, J. R., Rowe, J. B., Cusack, R., Calder, A. J., Marslen-Wilson, W. D., Duncan, J., Dalgleish, T., Henson, R. N., Brayne, C., & Matthews, F. E. (2014). The Cambridge Centre for Ageing and Neuroscience (Cam-CAN) study protocol: A cross-sectional, lifespan, multidisciplinary examination of healthy cognitive ageing. *BMC Neurology*, *14*(1), 204. 10.1186/s12883-014-0204-1 25412575 PMC4219118

[CIT0041] Shakeshaft, N. G., & Plomin, R. (2015). Genetic specificity of face recognition. *Proceedings of the National Academy of Sciences*, *112*(41), 12887–12892. 10.1073/pnas.1421881112 PMC4611634 26417086

[CIT0042] Sheppard, L. D., & Vernon, P. A. (2008). Intelligence and speed of information-processing: A review of 50 years of research. *Personality and Individual Differences*, *44*(3), 535–551. 10.1016/j.paid.2007.09.015

[CIT0043] Verhallen, R. J., Bosten, J. M., Goodbourn, P. T., Lawrance-Owen, A. J., Bargary, G., & Mollon, J. D. (2017). General and specific factors in the processing of faces. *Vision Research*, *141*, 217–227. 10.1016/j.visres.2016.12.014 28077292

[CIT0044] West, J. T., Horning, S. M., Klebe, K. J., Foster, S. M., Cornwell, R. E., Perrett, D., Burt, D. M., & Davis, H. P. (2012). Age effects on emotion recognition in facial displays: From 20 to 89 years of age. *Experimental Aging Research*, *38*(2), 146–168. 10.1080/0361073X.2012.659997 22404538

[CIT0045] Wilhelm, O., Herzmann, G., Kunina, O., Danthiir, V., Schacht, A., & Sommer, W. (2010). Individual differences in perceiving and recognizing faces – One element of social cognition. *Journal of Personality and Social Psychology*, *99*(3), 530–548. 10.1037/a0019972 20677889

[CIT0046] Young, A. W. (2018). Faces, people and the brain: The 45th Sir frederic Bartlett lecture. *Quarterly Journal of Experimental Psychology*, *71*(3), 569–594. 10.1177/1747021817740275 29461174

[CIT0047] Young, A. W., & Burton, A. M. (2018). Are we face experts? *Trends in Cognitive Sciences*, *22*(2), 100–110. 10.1016/j.tics.2017.11.007 29254899

[CIT0048] Young, A. W., Perrett, D., Calder, A., Sprengelmeyer, R., & Ekman, P. (2002). *Facial expressions of emotion: Stimuli and tests (FEEST)*. Thames Valley Test Company.

[CIT0049] Young, A. W., Rowland, D., Calder, A. J., Etcoff, N. L., Seth, A., & Perrett, D. I. (1997). Facial expression megamix: Tests of dimensional and category accounts of emotion recognition. *Cognition*, *63*(3), 271–313. 10.1016/S0010-0277(97)00003-6 9265872

