# Supplementary material for: Face perception across the adult lifespan: evidence for age-related changes independent of general intelligence
Source: Cogn Emot. 2021 Mar 18;35(5):890–901. doi: 10.1080/02699931.2021.1901657 (PMC8372290; doi:10.1080/02699931.2021.1901657)
Supplement: Supplementary_Material [file PCEM_A_1901657_SM3616.docx]

**Appendix**

Note, the correlation between anger and disgust was between the residuals of these two variables. The residuals are omitted here in the interest of visual clarity. Bolded values indicate significance at *p*<.05, and fit indices are presented below each model.

CFI: .96; RMSEA: .06; AIC: 42556.49

Anger

Disgust

Fear

Surprise

Happiness

Sadness

Face ID

Age

Sex

General Intelligence

**-.20**

**.16**

**.22**

**.45**

**.25**

**.62**

**.51**

**.71**

**.26**

**.63**

**.40**

**.26**

**Age -> Disgust model**

CFI: .96; RMSEA: .06; AIC: 42565.63

Anger

Disgust

Fear

Surprise

Happiness

Sadness

Face ID

Age

Sex

General Intelligence

-.09

**.16**

**.24**

**.49**

**-.17**

**.64**

**.33**

**.57**

**.27**

**.64**

**.41**

**.29**

**Age -> Fear model**

CFI: .95; RMSEA: .07; AIC: 42572.23

Anger

Disgust

Fear

Surprise

Happiness

Sadness

Face ID

Age

Sex

General Intelligence

**-.20**

**.15**

**.22**

**.46**

**.13**

**.62**

**.30**

**.70**

**.36**

**.63**

**.41**

**.31**

**Age -> Happiness model**

CFI: .95; RMSEA: .07; AIC: 42577.57

Anger

Disgust

Fear

Surprise

Happiness

Sadness

Face ID

Age

Sex

General Intelligence

**-.20**

**.15**

**.23**

**.46**

.04

**.62**

**.30**

**.70**

**.25**

**.66**

**.41**

**.31**

**Age -> Sadness model**

CFI: .95; RMSEA: .07; AIC: 42573.77

Anger

Disgust

Fear

Surprise

Happiness

Sadness

Face ID

Age

Sex

General Intelligence

**-.17**

**.15**

**.23**

**.46**

**-.11**

**.63**

**.31**

**.71**

**.26**

**.63**

**.32**

**.30**

**Age -> Surprise model**
